# Polycystin-1 Downregulation Induced Vascular Smooth Muscle Cells Phenotypic Alteration and Extracellular Matrix Remodeling in Thoracic Aortic Dissection

**DOI:** 10.3389/fphys.2020.548055

**Published:** 2020-09-24

**Authors:** Jing Zhang, Fei Liu, Yu-bin He, Wei Zhang, Wen-rui Ma, Jie Xing, Li-xin Wang

**Affiliations:** ^1^Department of Cardiovascular Surgery, Shanghai Chest Hospital, Shanghai Jiao Tong University, Shanghai, China; ^2^Department of Vascular Surgery, Zhongshan Hospital, Fudan University, Shanghai, China; ^3^Department of Surgery Base, Huashan Hospital North, Fudan University, Shanghai, China; ^4^Department of Cardiac Surgery, Zhongshan Hospital, Fudan University, Shanghai, China; ^5^Department of Biobank, Shanghai Chest Hospital, Shanghai Jiao Tong University, Shanghai, China; ^6^Department of Vascular Surgery, Xiamen Branch, Zhongshan Hospital, Fudan University, Shanghai, China

**Keywords:** polycystin-1, vascular smooth muscle cells, phenotype, aortic dissection, extracellular matrix, rapamycin

## Abstract

**Objective:**

Polycystin-1 (PC-1) is a protein encoded by the gene of polycystic kidney disease-1 (PKD-1). This study was designed to investigate the regulatory mechanisms of PC-1 on phenotypes of aortic vascular smooth muscle cells (VSMCs) and functions of extracellular matrix (ECM) in thoracic aortic dissection (TAD).

**Methods:**

Aortic tissues from patients with TAD and healthy controls were collected, primary aortic VSMCs were also isolated. Immunohistochemistry, immunofluorescence, and immunocytochemistry was used to visualize the target proteins. Western blot and RT-qPCR were used to examine the expression of mRNA and proteins. Lentivirus infection was used to downregulate or overexpress PC-1.

**Results:**

Compared with the control group, expression of PC-1 and the contractile phenotypic markers of VSMCs were decreased in TAD group, whereas expression of the synthetic markers of VSMCs, matrix metalloproteinase (MMP)-2, collagen I and collagen III were increased. The phosphorylation of mTOR, S6K and S6 were also elevated in TAD group. PC-1 downregulation of aortic VSMCs inhibited the expression of the contractile markers, but elevated the expression of the synthetic markers, MMP-2, collagen I and collagen III compared with the control group. The phosphorylation of mTOR, S6K and S6 were also increased in PKD-1-knockdown VSMCs. PC-1 upregulation reversed all these expression characteristics in aortic VSMCs. Furthermore, rapamycin treatment to PKD-1-knockdown VSMCs inhibited the effects caused by PC-1 downregulation.

**Conclusion:**

Our study revealed PC-1 downregulation induces aortic VSMCs phenotypic alteration and ECM remodeling via activation of mTOR/S6K/S6 signaling pathway. Downregulation of PC-1 might be a potential mechanism for the development and progression of TAD. Rapamycin might be a potential inhibitor to attenuate the development and progression of TAD.

## Introduction

Thoracic aortic dissection (TAD) is characterized by the tear of aortic intima and the false lumen in aortic media (Wang et al., 2006; Nienaber et al., 2016), which is a fatal condition with the risks of aortic rupture and malperfusion of branches. In recent years, the prevalence of TAD has been increasing all over the world (Howard et al., 2013; Landenhed et al., 2015). Based on aortic location, TAD can be clinically classified into Stanford type A (ascending aorta) and Stanford type B (descending aorta). Although endovascular repair has greatly improved the surgical outcomes of Stanford type B TAD, open surgery is still the only effective treatment for Stanford type A TAD with high mortality and severe complications. Thus, it is urgent to illustrate the potential mechanisms for TAD whereby contributing to the advance of clinical practice.

Extracellular matrix (ECM) dysfunction in aortic media is the common histological feature of TAD (Wang et al., 2006). The aortic media is comprised of vascular smooth muscle cells (VSMCs) and ECM (Amabili et al., 2019). As the most abundant ECM components in the aortic wall, elastin and collagen are responsible for aortic mechanical properties. VSMCs are the main source of ECM in the aortic media, including two different functional conditions, namely, the contractile and the synthetic phenotype. The contractile VSMCs are usually characterized by the weak proliferation, the attenuated migration and high expression of the contractile genes. By contrast, the synthetic VSMCs exhibit the enhanced proliferation, migration and ECM synthesis (Wang Y. et al., 2019). The phenotypic alteration between contractile and synthetic VSMCs is indispensable for the maintenance of aortic homeostasis. However, the pathological phenotypic alteration may play a pivotal role in the pathogenesis of TAD.

Polycystin (PC)-1 is a transmembrane protein encoded by the gene of polycystic kidney disease-1 (PKD-1), which is firstly recognized as a regulator for autosomal-dominant polycystic kidney disease (ADPKD) (Winokurow and Schumacher, 2019). The mutations of PKD-1 and the downregulation of PC-1 have been proved to be associated with renal cyst formation, as well as abnormal epithelial cell proliferation, cell adhesion and cell-matrix communication (Papavassiliou et al., 2019). Notably, the stable expression of PC-1 is essential for VSMCs development in the process of embryogenesis (Boulter et al., 2001; Qian et al., 2003). However, the role of PC-1 in the pathogenesis of TAD remains unclear.

In the present study, we regulated the expression of PC-1 to observe the alterations of aortic VSMCs phenotypes and ECM components. The purpose of this study was to explore the potential mechanisms in the regulation of PC-1 on VSMCs phenotypic alteration and ECM remodeling.

## Materials and Methods

### Collection of Aortic Samples

The collection and use of human aortic samples were approved by the Ethical Committee of Shanghai Chest Hospital. Twenty-one ascending aortic samples were collected from patients with acute Stanford type A TAD who have underwent the surgical procedures. Patients diagnosed with bicuspid aortic valve, Marfan Syndrome, Ehlers-Danlos syndrome, familial thoracic aortic aneurysm and dissection, chronic TAD and acute Stanford Type A intramural hematoma were excluded from the study cohort. Twelve normal ascending aortic samples were donated by the donors for heart transplantation without cardiovascular diseases. All patients’ and donors’ information were available. Patient demographics were listed in Supplementary Table 1.

### Histopathology

All specimens were fixed with 4% paraformaldehyde (PFA) at 4°C overnight, embedded with paraffin and 4 μm sections were cut. All sections were subjected to antigen retrieval, endogenous peroxidase was blocked with 3% hydrogen peroxide, and non-specific binding sites were blocked with10% goat serum at room temperature for 1 h. All slides were incubated with primary antibodies overnight at 4°C. Then, the slides were incubated with secondary antibodies at room temperature for 1 h. 3, 3-diaminobenzidine (DAB) (K5007, Dako, Denmark) was added for 10 min to visualize the proteins *in situ.* All pictures were captured by an optical microscope (Leica, Germany). The positive areas of slides were analyzed by Image-Pro Plus 6.0 (Media Cybernetics, United States) and presented as the relative value of IOD sum/Area sum. In this study, osteopontin (OPN) and myosin heavy chain (MYH)10 were served as the synthetic phenotypic markers of aortic VSMCs (Milewicz et al., 2017). Smooth muscle actin-22α (SM22α) and calponin were chosen as the contractile markers (Xu et al., 2019).

### Von Gienson and Masson’s Trichrome Staining

All specimens were fixed with 4% PFA overnight at 4°C, embedded with paraffin and 4 μm sections were cut. The elastic fiber and collagen staining were performed by using an elastic staining kit (Sigma-Aldrich, HT25A) and Masson’s Trichrome Staining Kit (Solarbio, G1340, Beijing, China). All procedures were performed according to the manufacturer’s instruction.

### Isolation of Human Aortic VSMCs

The explanted aorta was stored in PBS supplemented with 3% penicillin/streptomycin. After transferred to laboratory, the aortic tissues were washed by PBS for 3 times to remove the residual red blood cells. After stripping the intima and adventitia, the media was cut into 1-mm^2^ explants and digested for 3 h (Normal aorta) or 1 h (TAD aorta) in an enzymatic mix containing 2.5% collagenase type II and 1 mg/ml elastase. Then the mix was centrifugated, resuspended with Dulbecco’s Modified Eagle Medium (DMEM, Gibco, United States) supplemented with 10% fetal bovine serum (FBS), 1% penicillin/streptomycin and Glutamax (35050061, Gibco, United States) and seeded into T25 flask. The medium was changed every 2 days and cells between passage 2 to 4 were used for lentiviral infection or rapamycin treatment.

### Immunocytochemistry (ICC)

Cells in six-well plates were fixed with 4% PFA for 30 min at room temperature and washed with phosphate buffer saline (PBS). 10% goat serum was used to block non-specific binding sites. Then primary antibodies were incubated with the samples overnight at 4°C. The primary antibodies were removed by washing, and the slides were incubated with secondary antibodies at room temperature for 1 h and subsequently stained with 4′,6-diamidino-2-phenylindole (DAPI) (KGA-215, Keygen, China) for 2 min or DAB for 10 min. The target proteins were visualized with laser confocal microscope (Leica, Germany) or optical microscope.

### Lentiviral Infection

The PKD-1 short hairpin RNA (shRNA) expression plasmids, the subcloned full length cDNA for PKD-1 plasmids, the negative control (NC) plasmids and lentiviral packaging plasmids were amplified by standard bacterial transformation techniques and purified by a CompactPrep Plasmid Midi Kit (12843, QIAGEN, Germany). Lentiviruses that expressed PKD-1 shRNA, subcloned full length cDNA of PC-1 and NC were generated by co-transfection of the plasmids Helper 1.0 and Helper 2.0 into 293T cells. After 72 h, the lentiviral supernatants were collected and purified. Normal aortic VSMCs were infected with lentivirus expressing PKD-1 shRNA, subcloned full length cDNA of PC-1 and NC in the presence of polybrene. The expression of GFP was examined by a fluorescence microscope (Zeiss, Germany) after 72 h.

### Real-Time Reverse Transcription Quantitative Polymerase Chain Reaction (RT-qPCR)

Total RNA of cultured cells was extracted by a Trizol (Invitrogen, United States) reagent and converted to cDNA by a Hitranscript 1st Strand Synthesis Kit (R-211, Vazyme, China). The PCR procedure was performed using ChamQ SYBR Color qPCR Master Mix (Q321, Vazyme, China) in a StepOne Plus System (Life Technologies, United States). The primers for the RT-qPCR were synthesized by BGI company (Shenzhen, China). All primers were listed in Supplementary Table 2. The mRNA expression was standardized to glyceraldehyde-3-phosphate dehydrogenase (GAPDH).

### Western Blot Analysis

Total protein from cultured cells, normal and pathologic aortic tissues was extracted by the RIPA cell lysis reagent (Sigma, United States) on the ice for 30 min. Equivalent amounts of proteins were separated via SDS-PAGE and transferred onto 0.45 μm polyvinylidene difluoride (PVDF) membranes. Then the membranes were blocked with 5% non-fat dry milk for 1 h at room temperature. After blockage, the membranes were incubated at 4°C overnight with a rabbit anti-osteopontin (OPN)antibody (ab8448, Abcam, United Kingdom), a goat anti-SM22α antibody (ab10135, Abcam, United Kingdom), a mouse anti-PC-1 antibody (NBP2-50247, Novus, United States), a mouse anti-calponin antibody (ab700, Abcam, United Kingdom), a mouse anti-myosin heavy chain 10 (MYH10) antibody (ab684, Abcam, United Kingdom), a rabbit anti-collagen I antibody (ab34710, Abcam, United Kingdom), a mouse anti-collagen III antibody (ab23445, Abcam, United Kingdom), a rabbit anti-matrix metalloproteinase (MMP)-1 antibody (54376S, CST, United States), a rabbit anti-MMP2 antibody (40994S, CST, United States), a rabbit anti-mTOR antibody (2983S, CST, United States), a rabbit anti-phosphorylated-mTOR antibody (2974S, CST, United States), a rabbit anti-S6 ribosomal protein kinase (S6K) antibody (2708T, CST, United States), a rabbit anti-phosphorylated-S6K antibody (9205S, CST, United States), a mouse anti-S6 ribosomal protein (S6) antibody (9205S, CST, United States), a rabbit anti-phosphorylated-S6RP antibody (4858T, CST, United States), a rabbit anti-4E binding protein (4E-BP) antibody (9644T, CST, United States), a rabbit anti-phosphorylated-4E-BP antibody (2855T, CST, United States) and a rabbit anti-eukaryotic initiation factor 4E (EIF4E)antibody (2067T, CST, United States). Then the membranes were washed for three times and incubated with secondary antibodies at room temperature for 1h. The immune complexes were visualized by an ECL Kit (Millipore, United States) and images were captured. The quantification of proteins was standardized by GAPDH and analyzed by Image J (NIH, United States).

### Enzyme Linked Immunosorbent Assay (ELISA)

The secretion of collagens was detected by collagen I (E4617-100, Biovision, United States) and collagen III (DL-COL3-Hu-48T, Dldevelop, United States) ELISA kits according to the manufacturer’s instructions.

### Cell Viability and Proliferation Assay

Cells were seeded into 96-well plates at a density of 1 × 10^4^ cells. The cell viability and proliferation were detected at 0, 24, 48, 72, and 96 h. The CCK-8 (Dojindo, Japan) reagent was added into 96-well plates and incubated for 1 h. Then the absorbance at 450 nm and was detected by a microplate reader (Gen5, BioTek, United States).

### Wound Healing Assay

Cells were seeded into 6-well plates at a density of 2 × 10^5^ cells. When the cells confluenced to 80% and monolayer was generated, a 200 μl pipette tip was utilized to form three separate wounds. Subsequently, cells were washed and allowed to move up to 24 h. The images at 0, 6, 12, and 24 h were captured by an inverted phase contrast microscopy (A480, Zeiss, Germany). Further analysis was performed by Image J.

### Transwell Assay

Cells were seeded into the upper compartment of the modified 12-well transwell system chamber (Corning, United States) at the density of 1 × 10^5^ cells. Serum-free medium was added into the upper chamber and the culture medium was added into the lower chamber. After 24 h, cells that migrated to the lower compartment were fixed with 4% PFA for 30 min at room temperature and stained with 1% crystal violet for 20 min. The stained cells were counted by Image J at five random areas.

### Statistical Analysis

The data were analyzed by SPSS 20.0 (Chicago, United States) and presented as the mean ± standard deviation of six independent experiments. Student’s *t*-test, one-way ANOVA and followed the Dunnett test, linear regression and correlation analysis were used to analyze the data. Differences were considered statistically significant when *p* < 0.05.

## Results

### Reduced PC-1 Expression Was Related to VSMCs Phenotypic Transition in TAD Group

**FIGURE 1:**
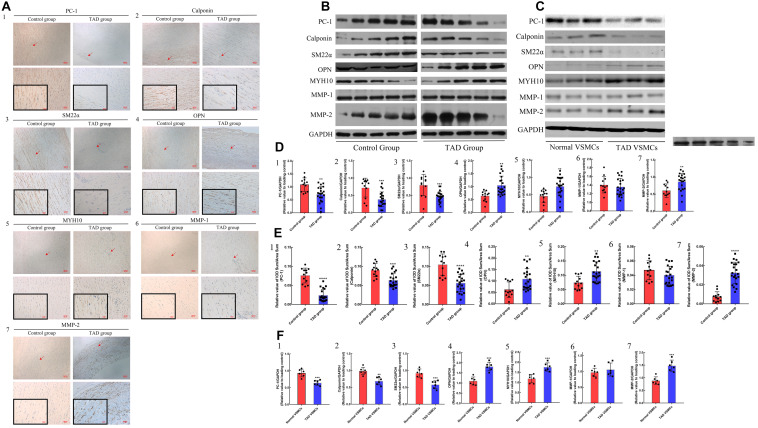
IHC results and protein expression in control (*n* = 12) and TAD group (*n* = 21) and *in vitro* studies between normal (*n* = 6) and TAD VSMCs (*n* = 6). Decreased positive area of **(A1,E1)** PC-1 (*p* < 0.0001), **(A2,E2)** calponin (*p* = 0.0002) and **(A3,E3)** SM22α (*p* < 0.0001) as well as increased positive area of, **(A4,E4)** OPN (*p* = 0.0049), **(A5,E5)** MYH10 (*p* = 0.0011) and **(A7,E7)** MMP-2 (*p* < 0.0001) in the TAD group. The positive area of **(A6,E6)** MMP-1 showed no significance between two groups. Increased expression of **(B,D4)** OPN (*p* = 0.001), **(B,D5)** MYH10 (*p* = 0.0029), **(B,D7)** MMP-2 (*p* = 0.0034) as well as decreased expression of **(B,D1**) PC-1 (*p* = 0.0011), **(B,D2)** calponin (*p* = 0.0004) and **(B,D3)** SM22α (*p* = 0.0002) in TAD group. The expression of **(A)** MMP-1 were not significant between two groups. TAD VSMCs exhibited lower expression of **(C,F1–3)** PC-1 (*p* = 0.0004), calponin (*p* = 0.001) and SM22α (*p* = 0.0005) as well as higher expression of **(C,F4,5,7)** OPN (*p* = 0.0001), MYH10 (*p* = 0.0008), MMP-2 (*p* = 0.0003), but the **(C,F6)** MMP-1 were not significantly different between these groups. **p < 0.01, ***p < 0.001, ****p < 0.0001.

In order to investigate the expression of PC-1 and the relationship between the level of PC-1 and the phenotype of VSMCs in control group and TAD group, IHC, RT-qPCR and western blot were performed. The IHC exhibited decreased expression of PC-1 in TAD group (Figures 1A1,E1; *p* < 0.0001). The positive area of calponin and SM22α also presented lower levels in TAD group (Figures 1A2,3,E2,3; *p* < 0.01 for both). On the contrary, the positive area of OPN and MYH10 were much higher in TAD group (Figures 1A4,5,E4,5; *p* < 0.01 for both). The protein levels were also similar to the IHC results (Figures 1B,D1–5; *p* < 0.01 for all). PC-1 downregulation in TAD was accompanied by the elevated expression of MMP-2 (Figures 1A7,B,D7,E7; *p* < 0.01 for both). However, the expression of MMP-1 showed no significant differences between control and TAD groups (Figures 1A6,B,D7,E6; *p* > 0.05 for both).

The *in vitro* studies also showed reduced expression of PC-1, calponin and SM22α as well as elevated expression of OPN, MYH10 and MMP-2 in TAD VSMCs (Supplementary Figure 1A–E,G and Figures 1C,F1–5,7; *p* < 0.05 for all). The expression of MMP-1 did not exhibit difference between normal and TAD VSMCs (Supplementary Figure 1F and Figures 1C,F6; *p* > 0.05 for both). ICC also implied abated fluorescence intensity of SM22α and enhanced intensity of OPN in TAD VSMCs compared with normal VSMCs (Figures 6A1,2,B,C1,2,D; *p* < 0.05 for both). These results demonstrated the reduced expression of PC-1 was related to the phenotypic alteration from contractile VSMCs to synthetic VSMCs.

### Decreased PC-1 Expression Was Associated With ECM Dysfunction in TAD Group

**FIGURE 2:**
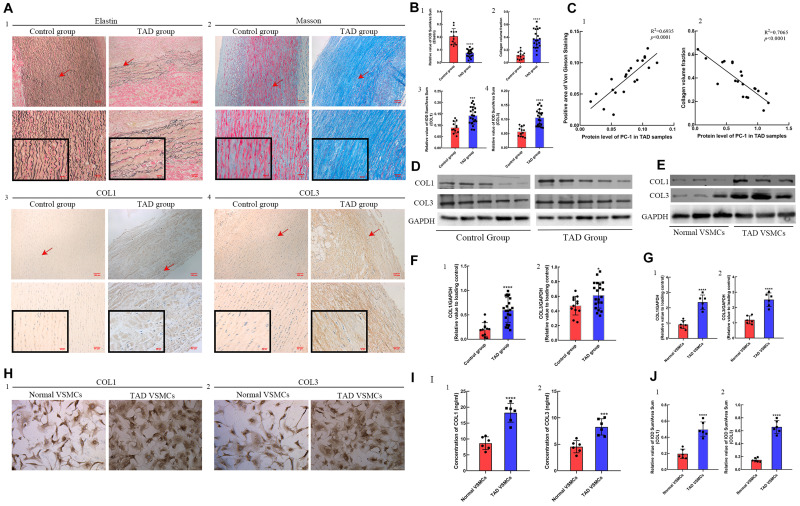
ECM staining in control (*n* = 12) and TAD group (*n* = 21) and protein expression between normal and TAD VSMCs. Decreased positive area of **(A1,B1)** von Gienson elastin staining in TAD group (*p* < 0.0001). **(A2,B2)** Increased collagen volume fraction (*p* < 0.0001), positive area of **(A3,B3)** collagen I (*p* = 0.0002) and **(A4,B4)** collagen III (*p* < 0.0001) in TAD group. The PC-1 expression was **(C1)** positively correlated with the positive area of von Gienson elastin staining (*R*^2^ = 0.6935, *p* < 0.0001) and **(C2)** negatively correlated with the collagen volume fraction (*R*^2^ = 0.7065, *p* < 0.0001) in TAD samples. Increased synthesis of **(D,F1)** collagen I (*p* < 0.0001) and **(D,F2)** collagen III (*p* = 0.0199) in TAD group. The synthesis of **(E,F1)** collagen I (*p* = 0.0007, *p* < 0.0001) and **(E,F2)** collagen III (*p* = 0.0019, *p* < 0.0001) was increased in TAD VSMCs. **(G1,2)** These pictures showed the protein expression of COL1 and COL3 between normal and TAD VSMCs. The secretion and deposition of **(I1,H1,J1)** collagen I (*p* < 0.0001, *p* < 0.0001) and **(I2,H2,J2)** collagen III (*p* = 0.0008, *p* < 0.0001) were increased in TAD VSMCs. ***p < 0.001, ****p < 0.0001.

In order to detect the fragmentation of elastic fiber and accumulation of collagens between control group and TAD group, Von Gienson and Masson’s trichrome staining were performed. TAD group showed fragmentation and decrease of elastic fibers (Figures 2A1,B1, *p* < 0.0001), together with increased deposition of collagen I and collagen III (Figures 2A2–4,B2–4; *p* < 0.001 for all). The correlation analysis indicated the positive correlation between PC-1 level and elastic fibers integrity (Figure 2C1, *R*^2^ = 0.6935, *p* < 0.0001) as well as the negative correlation between PC-1 level and collagen deposition (Figure 2C2, *R*^2^ = 0.7065, *p* < 0.0001). The protein levels of collagen I and collagen III in control and TAD groups were similar to the IHC results (Figures 2D,F1,2; *p* < 0.05 for both). Similarly, *in vitro* studies presented higher synthesis (Figures 2E,G1,2, *p* < 0.0001 for both), secretion (Figure 2J1,2, *p* < 0.0001 for both) and deposition (Figures 2H1,2,I1,2, *p* < 0.001 for both) of collagen I and collagen III in TAD VSMCs. These results showed that decreased expression of PC-1 was also related to the fragmentation of elastic fibers and over-accumulation of collagens, which led to the increased stiffness of aortic wall whereby promoting the development of TAD.

### PC-1 Downregulation Promoted Phenotypic Alteration From Contractile VSMCs to Synthetic VSMCs

**FIGURE 3:**
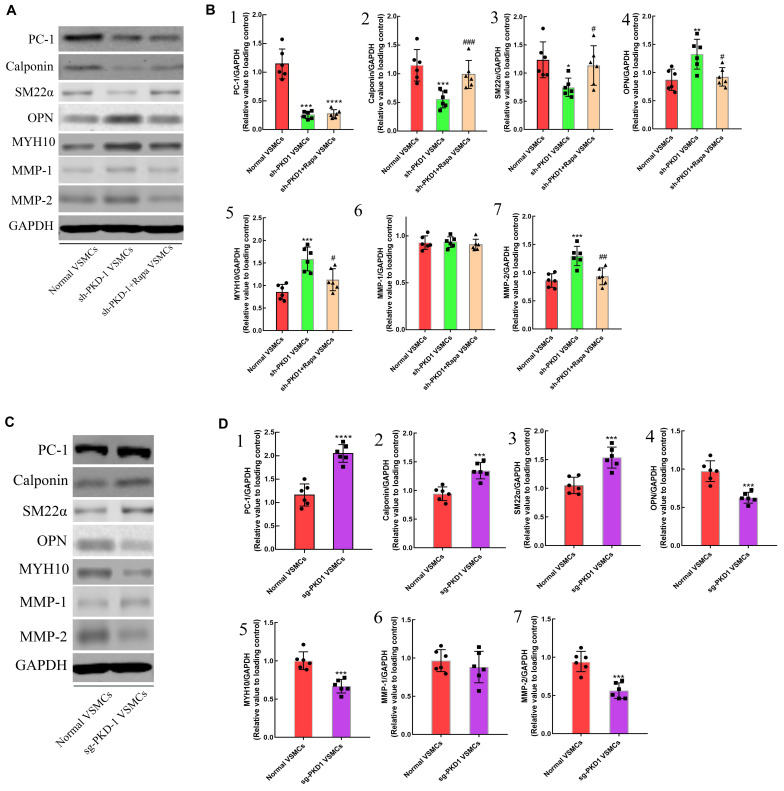
The effects of downregulation and overexpression of PKD-1 on the phenotypes of aortic VSMCs (*n* = 6 for each group). **(A)** PKD-1 knockdown inhibited the expression of **(A,B1)** PC-1 (*p* < 0.0001, *p* < 0.0001), **(A,B2)** calponin (*p* = 0.0001, *p* = 0.0008) and **(A,B3)** SM22α (*p* = 0.0053, *p* = 0.0194) as well as elevated the expression of **(A,B4)** OPN (*p* < 0.0001, *p* = 0.0037), **(A,B5)** MYH10 (*p* < 0.0001, *p* = 0.0001), **(A,B7)** MMP-2 (*p* < 0.0001, *p* = 0.0003) Rapamycin didn’t affect the expression of **(A,B1)** PC-1 (*p* < 0.0001, *p* < 0.0001) in sh-PKD-1 VSMCs but elevated the expression of **(A,B2)** calciponin (*p* = 0.0026, *p* = 0.0038) and **(A,B3)** SM22α (*p* = 0.0352, *p* = 0.034) and downregulated the expression of **(A,B4)** OPN (*p* = 0.0009, *p* = 0.0101), **(A,B5)** MYH10 (*p* = 0.0002, *p* = 0.0105), **(A,B7)** MMP2 (*p* < 0.0001, *p* = 0.0031). **(A,B6)** Showed the protein expression of MMP-1 among three groups. Treatment of sg-PKD-1 enhanced the expression of **(C,D1)** PC-1 (*p* < 0.0001, *p* < 0.0001), **(C,D2)** calciponin (*p* = 0.0054, *p* = 0.0004) and **(C,D3)** SM22α (*p* = 0.0005, *p* = 0.0003), but inhibited the expression of **(C,D4)** OPN (*p* = 0.0125, *p* = 0.0002), **(C,D5)** MYH10 (*p* = 0.0078, *p* = 0.0002), **(C,D7)** MMP2 (*p* < 0.0001, *p* = 0.0009). **(C,D6)** Showed the protein expression of MMP-1between normal and sg-PKD-1 VSMCs. *p < 0.05, **p < 0.01, ***p < 0.001, ****p < 0.0001 (compared with normal VSMCs); ##*p* < 0.01, ###*p* < 0.001 (compared with TAD VSMCs). ^#^Means the comparison between sh-PKD1 and sh-PKD1 + Rapa VSMCs. *p* < 0.05.

To further examine the effects of PC-1 downregulation to the phenotype of VSMCs, lentivirus infection was used to downregulate or overexpress the PC-1 in human aortic primary VSMCs. RT-qPCR and western blot were used to detect the expression of mRNA and protein levels of related genes and proteins. PKD-1 knockdown inhibited the expression of PC-1, calponin and SM22α (Supplementary Figures 2A1–3 and Figures 3A,B1–3, *p* < 0.05 for all), and enhanced the expression of OPN, MYH10 and MMP-2 in sh-PKD-1 VSMCs (Supplementary Figures 2A4,5,7, Figures 3A,B4,5,7; *p* < 0.05 for all). PKD-1 knockdown did not affect MMP-1 expression (Supplementary Figure 2A6 and Figures 3A,B6; *p* > 0.05 for both). ICC revealed decreased fluorescence intensity of SM22α and increased intensity of OPN in sh-PKD-1 VSMCs compared with normal and TAD VSMCs (Figures 6A3,B,C3,D; *p* < 0.01 for all).

In contrast, PKD-1 overexpression stimulated PC-1 expression (Supplementary Figure 2B1 and Figures 3C,D1, *p* < 0.05 for both), which correspondingly led to upregulation of calponin and SM22α (Supplementary Figure 2B2,3 and Figures 3C,D2,3, *p* < 0.05 for all) as well as downregulation of OPN, MYH10 and MMP-2 in sg-PKD-1 VSMCs (Supplementary Figure 2B4,5,7 and Figures 3C,D4,5,7; *p* < 0.05 for all). However, the expression of MMP-1 remained unchanged (Supplementary Figure 2B6 and Figures 3C,D6; *p* > 0.05 for both). ICC exhibited elevated fluorescence intensity of SM22α and diminished intensity of OPN in sg-PKD-1 VSMCs compared with normal VSMCs (Figures 6A5,B,C5,D; *p* < 0.05 for both).

### Diminished PC-1 Expression in Aortic VSMCs Induced Over-Deposition of Collagens

**FIGURE 4:**
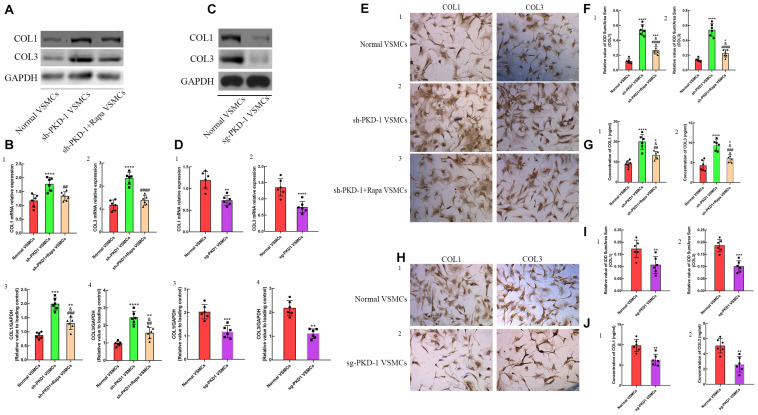
The synthesis, secretion and deposition of collagens when the expression of PC-1 was inhibited or enhanced. Increased synthesis of **(A,B1,3)** collagen I (*p* < 0.0001, *p* = 0.0003) and **(A,B2,4)** collagen III (*p* < 0.0001, *p* < 0.0001) in sh-PKD-1 VSMCs compared with normal VSMCs. Enhanced secretion of **(G1**) COL1 (*p* < 0.0001) and **(G2)** COL3 (*p* < 0.0001) in sh-PKD-1VSMCs compared with normal VSMCs. Increased deposition of COL1 (*p* < 0.0001) and COL3 (*p* < 0.0001) in sh-PKD-1 VSMCs compared with normal VSMCs. sh-PKD-1 + Rapa VSMCs exhibited suppressed synthesis of (**A,B1,3)** COL1 (*p* = 0.0044, *p* = 0.0003) and **(A,B2,4)** COL3 (*p* < 0.0001, *p* = 0.0014) compared with sh-PKD-1 VSMCs. **(E1)** Showed the ICC results of COL1 and COL3 in normal VSMCs. sh-PKD-1 + Rapa treatment also inhibited the **(G1,2**) secretion (*p* = 0.0031, *p* = 0.0006) and **(E2,3,F1,2)** deposition (*p* < 0.0001, *p* = 0.0006) of COL1 and COL3 compared with sh-PKD-1 VSMCs. Treatment of sg-PKD1 inhibited the synthesis of **(C,D1,3**) COL1 (*p* = 0.0006, *p* = 0.0004) and **(C,D2,4)** COL3 (*p* = 0.0009, *p* < 0.0001) compared with normal VSMCs. The **(J1,2)** secretion (*p* = 0.0015, *p* = 0.0017) and **(H1,2,I1,2)** deposition (*p* = 0.0088, *p* = 0.0001) of COL1 and COL3 were also suppressed in sg-PKD-1 VSMCs compared with normal VSMCs. *p < 0.05, **p < 0.01, ***p < 0.001, ****p < 0.0001 (compared with normal VSMCs); ##*p* < 0.01, ###*p* < 0.001 (compared with TAD VSMCs). ####Means the comparison between sh-PKD1 and sh-PKD1 + Rapa VSMCs. *p* < 0.0001.

To verify the influence of PC-1 expression to collagens deposition of VSMCs, the primary aortic VSMCs were cultured. RT-qPCR and western blot were used to detect the synthesis of collagens. ELISA was used to quantify the secreted collagens in the supernatant of cultured VSMCs. ICC was used to visualize the deposition of collagens. The expression of collagen I and collagen III were increased in sh-PKD-1 VSMCs (Figures 4A,B1–4; *p* < 0.01 for all), which indicated the augmented synthesis of collagens. The excessive secretion (Figure 4G1,2, *p* < 0.0001 for both) and deposition (Figures 4E1,2,F1,2, *p* < 0.0001 for both) of collagen I and collagen III were also found in sh-PKD-1 VSMCs. In contrast, PKD-1 overexpression suppressed the synthesis (Figures 4C,D1–4, *p* < 0.0001 for all), secretion (Figure 4J1,2, *p* < 0.01 for both) and deposition (Figures 4H1,2,I1,2, *p* < 0.01 for both) of collagen I and collagen III.

### Activation of mTOR/S6K/S6 Promoted Aortic VSMCs Phenotypic Alteration and Collagen Over-Deposition

**FIGURE 5:**
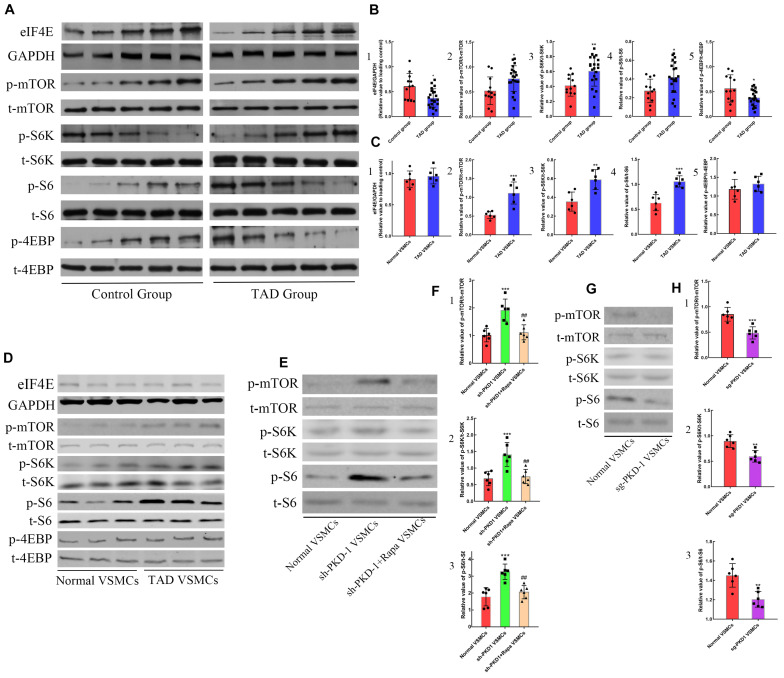
The effects of PC-1 expression on the signaling pathway in human samples and VSMCs. Increased expression of **(A,B1)** eIF4E (*p* = 0.0128) as well as phosphorylation of **(A,B2)** mTOR (*p* = 0.0159), **(A,B3)** S6K (*p* = 0.0066), **(A,B4)** S6 (*p* = 0.0126) and **(A,B5)** 4EBP (*p* = 0.0189) in TAD group compared with control group. **(C2–4,D)** The phosphorylation of mTOR (*p* = 0.0009), S6K (*p* = 0.0029) and S6 (*p* = 0.0004) were elevated in TAD VSMCs, but the phosphorylation of **(C1,D)** 4EBP and expression of **(C5,D)** eIF4E were not altered. The phosphorylation of **(E,F1)** mTOR (*p* = 0.0003), **(E,F2)** S6K (*p* = 0.0007) and **(E,F3)** S6 (*p* = 0.0001) was enhanced in sh-PKD-1 VSMCs compared with normal VSMCs. The phosphorylation of **(E,F1)** mTOR (*p* = 0.0022), **(E,F2)** S6K (*p* = 0.003) and **(E,F3)** S6 (*p* = 0.0006) were inhibited in sh-PKD-1 + Rapa VSMCs compared with sh-PKD-1 VSMCs. The phosphorylation of **(G,H1)** mTOR (*p* = 0.0006), **(G,H2)** S6K (*p* = 0.0015) and **(G,H3)** S6 (*p* = 0.002) were decreased in sg-PKD-1 VSMCs compared with the control group. ##*p* < 0.01, *p < 0.05, **p < 0.01, ***p < 0.001.

In order to demonstrate the regulative mechanism of PC-1-downregulation-induced phenotypic alteration of VSMCs, the inhibitor of mTOR signaling pathway was used to treat the sh-PKD-1 VSMCs. The phosphorylation of mTOR signaling pathway was detected via western blot. The phosphorylation of mTOR, S6K and S6 were elevated in TAD group (Figures 5A,B2–4; *p* < 0.05 for all). However, 4EBP in TAD group exhibited the diminished phosphorylation and further inhibited the expression of eIF4E (Figures 5A,B1,5; *p* < 0.05 for both).

*In vitro* studies showed the higher phosphorylation of mTOR, S6K and S6 in TAD VSMCs (Figures 5C2–4,D; *p* < 0.01 for all), but the phosphorylation of 4EBP and the expression of eIF4E showed no difference between normal and TAD VSMCs (Figures 5C1,5,D; *p* > 0.05 for both). PKD-1 knockdown also led to the higher phosphorylation of mTOR, S6K and S6 compared with normal VSMCs (Figures 5E,F1–3; *p* < 0.001 for all). On the contrary, PKD-1 overexpression significantly inhibited the phosphorylation of mTOR, S6K and S6 (Figures 5G,H1–3; *p* < 0.01 for all).

**FIGURE 6:**
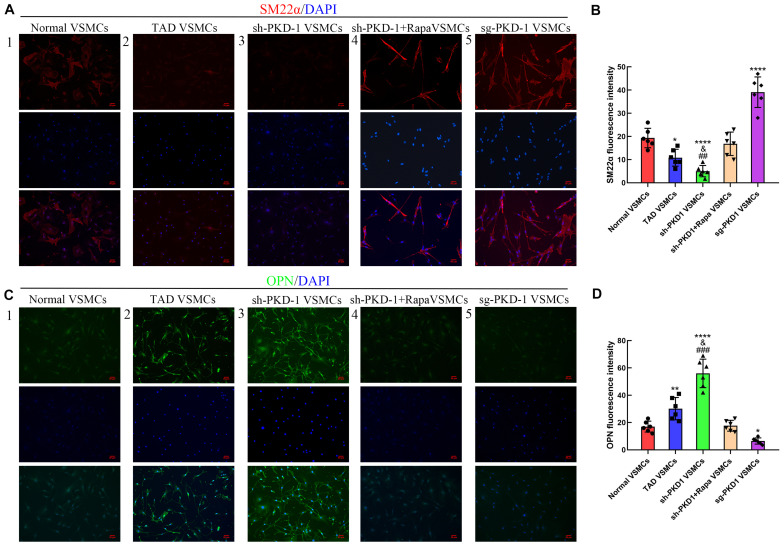
Immunofluorescence of SM22α and OPN among groups (*n* = 6 for each group). **(A,B)** The fluorescence intensity of SM22α was decreased in TAD VSMCs (*p* = 0.0128) and sh-PKD-1 VSMCs (*p* < 0.0001) but enhanced in the sg-PKD-1 group (*p* < 0.0001) compared with normal VSMCs. The intensity was increased in sh-PKD-1 VSMCs (*p* = 0.0088) compared with the TAD VSMCs, but showed no significance in sh-PKD-1 + Rapa VSMCs compared with normal VSMCs. **(C,D)**. The fluorescence intensity of OPN was elevated in TAD VSMCs (*p* = 0.0064) and sh-PKD-1 VSMCs (*p* < 0.0001) and decreased in sg-PKD-1 group (*p* = 0.034) compared with normal VSMCs. The intensity was enhanced in sh-PKD-1 VSMCs (*p* = 0.0008) compared with the TAD VSMCs, but showed no difference in sh-PKD-1 + Rapa VSMCs compared with normal VSMCs. *p < 0.05, **p < 0.01, ****p < 0.0001 (compared with normal VSMCs), ##*p* < 0.01, ###*p* < 0.001 (compared with TAD VSMCs).

When mTOR was blocked by rapamycin, the phosphorylation of mTOR/S6K/S6 in sh-PKD-1 + Rapa VSMCs were significantly lower than sh-PKD-1 VSMCs (Figures 5E,F1–3; *p* < 0.01 for all). Consistently, the expression of OPN, MYH10 and MMP-2 in sh-PKD-1 + Rapa VSMCs were much lower than sh-PKD-1 VSMCs (Supplementary Figures 2A4,5,B7 and Figures 3A,B4,5,7; *p* < 0.05 for all). However, the expression of calponin and SM22α in sh-PKD-1 + Rapa VSMCs were much higher than sh-PKD-1 VSMCs (Supplementary Figures 2A2,3 and Figures 3A,B2,3; *p* < 0.05 for all). The expression of MMP-1 was not affected by rapamycin treatment (Supplementary Figure 2A6 and Figures 3A,B6; *p* > 0.05 for all). Immunofluorescence also exhibited increased fluorescence intensity of SM22α and decreased fluorescence intensity of OPN, which were similar to the fluorescence intensity in normal VSMCs (Figures 6A4,B,C4,D, *p* > 0.05 for all). Rapamycin also potently inhibited the synthesis (Figures 4A,B1–4, *p* < 0.01 for all), secretion (Figure 4G1,2, *p* < 0.01 for both) and deposition (Figures 4E1,3,F1,2, *p* < 0.0001 for both) of collagen I and collagen III compared with sh-PKD-1 VSMCs.

### PC-1 Downregulation Promoted the Migration and Proliferation of Aortic VSMCs

**FIGURE 7:**
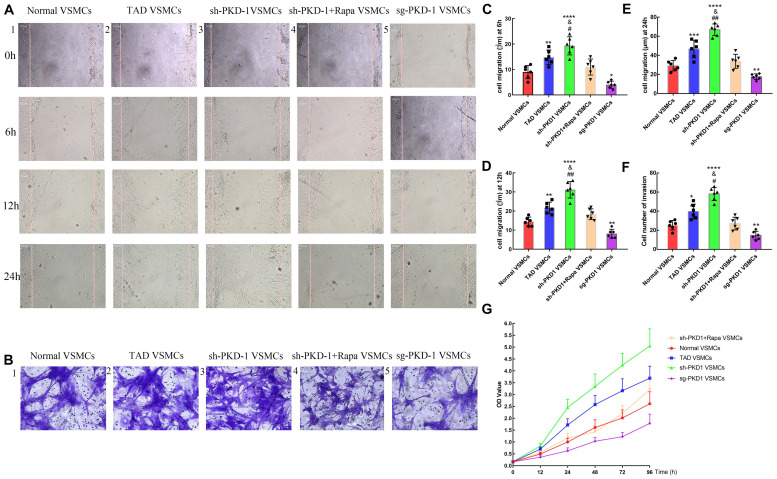
Cell migration and proliferation among groups (*n* = 6 for each group). **(A1–5)** Wound healing assay and **(B1–5)** transwell assay showed that cell migration was increased at **(C**) 6 h, **(D)** 12 h, and **(E,F)** 24 h in TAD VSMCs (*p* = 0.0051, *p* = 0.0016, *p* = 0.0004, *p* = 0.0021) and sh-PKD-1 VSMCs (*p* < 0.0001, *p* < 0.0001, *p* < 0.0001, *p* < 0.0001), but inhibited in **(C–F)** sg-PKD-1 group (*p* = 0.0174, *p* = 0.0051, *p* = 0.0191, *p* = 0.0021) compared with normal VSMCs. Cell migration was enhanced at these points **(C–F)** in sh-PKD-1 VSMCs (*p* = 0.0364, *p* = 0.0013, *p* = 0.0012, *p* = 0.0216) compared with TAD VSMCs. After treatment of rapamycin to sh-PKD-1 VSMCs, cell migration showed no significance compared with normal VSMCs. **(G)** Proliferation of VSMCs was enhanced at 12, 24, 48, 72, and 96 h in TAD VSMCs and sh-PKD-1 VSMCs, but mitigated in sg-PKD-1 group compared with normal VSMCs. Cell proliferation was promoted at these points in sh-PKD-1 VSMCs compared with TAD VSMCs. *p < 0.05, **p < 0.01, ***p < 0.001, ****p < 0.0001 (compared with normal VSMCs); #*p* < 0.05, ##*p* < 0.01 (compared with TAD VSMCs).

The wound healing assay and transwell assay were used to examine the migration of VSMCs. CCK8 assay was used to examine the proliferation of VSMCs among groups. The wound healing assay and transwell assay exhibited elevated migration at 6, 12, and 24 h in TAD VSMCs compared with normal VSMCs (Figures 7A2,B2,C–F; *p* < 0.05 for all). PKD-1 knockdown further enhanced the migration of VSMCs at 6, 12 and 24 h, which was much higher than normal and TAD VSMCs (Figures 7A3,B3,C–F; *p* < 0.05 for all). Otherwise, PKD-1 overexpression potently inhibited VSMCs migration at 6, 12, and 24 h (Figures 7A5,B5,C–F; *p* < 0.05 for all).

Cell proliferation among groups also presented similar tendency to the cell migration. Cell proliferation among groups exhibited no significant difference at 0 h. However, TAD VSMCs exhibited higher proliferation at 12, 24, 48, 72, and 96 h (Figure 7G and Supplementary Figures 3A–F; *p* < 0.01 for all). Otherwise, VSMCs in sh-PKD-1 VSMCs showed prominent proliferation at 12, 24, 48, 72, and 96 h compared with normal and TAD VSMCs (Figure 7G and Supplementary Figures 3A–F; *p* < 0.05 for all). Notably, sg-PKD-1 effectively inhibited the proliferation of VSMCs at 12, 24, 48, 72, and 96 h compared with normal VSMCs (Figure 7G and Supplementary Figures 3A–F; *p* < 0.05 for all).

Treatment of rapamycin to sh-PKD-1 VSMCs mitigated the cell migration, which was similar to normal VSMCs at 6, 12, and 24 h (Figures 7A4,B4,C–F; *p* > 0.05 for all). Proliferation of VSMCs after rapamycin treatment were also inhibited at 12, 24, 48, 72 and 96 h, which showed no significance compared with normal VSMCs (Figure 7G and Supplementary Figures 3A–F; *p* > 0.05 for all).

## Discussion

Through *in vitro* experiments, the present study has demonstrated that PC-1 downregulation, VSMCs phenotypic alteration and ECM dysfunction are involved in TAD. The present study is also novel to indicate that PC-1 downregulation induces aortic VSMCs phenotypic alteration and ECM dysfunction via activation of mTOR/S6K/S6 signaling pathway. Moreover, rapamycin treatment inhibited the effects caused by PC-1 on VSMCs phenotypes and ECM functions.

In physiological situations, the stable composition of ECM proteins with a low turnover rate is essential for maintaining the mechanical properties of the aortic wall. As shown in the present and previous studies (Wang et al., 2012; Wu et al., 2013; Zhang et al., 2013), severe elastic fiber degradation, massive collagen deposition, and elevated expression of MMPs are presented in TAD. The excessive and disorderly deposition of collagens increases aortic stiffness, and the disruption of elastic fibers leads to the decreased elasticity of aortic wall (Wagenseil et al., 2005; Wang et al., 2005), thus predisposing to TAD (Pozzi et al., 1998; Wang et al., 2006). ECM proteins and MMPs are produced by the synthetic VSMCs. MMP-1 (interstitial collagenase) degrades type I, II, and III collagens, while MMP-2 (gelatinase A) primarily degrades type IV collagen and elastin (Ishii and Asuwa, 2000; Borges et al., 2009). Therefore, the aberrant VSMCs phenotypic alteration might upset the subtle balance among ECM, MMPs, and VSMCs in the aortic wall, whereby contributing to the pathogenesis of TAD. The pathological phenotypic alteration has been reported to play an important role in the pathogenesis of aortic diseases (Xu et al., 2019). However, it remains unknown how to initiate VSMC phenotypic alteration in TAD.

PC-1 is a key factor in the regulation of several signaling pathways including STAT6 (signal transducer and activator of transcription 6) and mTOR (mammalian target of rapamycin) to maintain the homeostasis and differentiation of renal tubular epithelial cells (Verschuren et al., 2018; Tang et al., 2019). Notably, PC-1 is also involved in the pathogenesis of several cardiovascular diseases. The abated expression of PC-1 leads to the impaired contractility of cardiomyocytes via diminishing the calcium transients and sarcoplasmic reticulum calcium storage, consequently, resulting in cardiac dysfunction (Balbo et al., 2016; Altamirano et al., 2019). Deficiency of PC-1 in endothelial cells also exhibited alterations in mechanosensitivity which further affected the flow-mediated vascular dilation (Lorthioir et al., 2015). Recently, a study proposed that PC-1 might be a potential biomarker for early diagnosis of TAD, which was verified by the DNA microarray analysis of TAD specimens (Peng et al., 2015; Zhang et al., 2016). However, the regulatory effects of PC-1 on VSMCs and ECM have not been thoroughly elucidated. In this study, we found the expression of PC-1 was decreased in TAD. In addition, PC-1 downregulation promoted aortic VSMCs switching from contractile to synthetic type, together with the enhanced synthesis, secretion and deposition of collagen I and collagen III. In contrast, PC-1 upregulation reversed all these effects in aortic VSMCs. These results implied that PC-1 might be a negative regulator in the process of VSMCs phenotypic alteration in TAD, as well as exerting the protective effects to inhibit ECM dysfunction.

The activation of mTOR signaling pathway can stimulate VSMCs phenotypic alteration from contractile to the synthetic type (Pankratz et al., 2018). As the specific inhibitor of mTOR, rapamycin has been applied in clinical treatment of atherosclerosis to suppress VSMCs proliferation and migration. Several studies also found that rapamycin prevented the progression of aortic aneurysm by inhibiting VSMCs phenotypic alteration via PI3K/Akt/mTOR and PTEN/Akt/mTOR signaling pathway (Li et al., 2017; Peng et al., 2018; Wang Z. et al., 2019; Zhou et al., 2019). The present study demonstrated that the phosphorylation activation of mTOR/S6K/S6 was associated with VSMCs phenotypic alteration and ECM dysfunction in the pathogenesis of TAD. Furthermore, the activation of mTOR/S6K/S6 was regulated by PC-1 and inhibited by rapamycin. These findings indicate that, mTOR might be a potential target molecule in the pathogenesis of TAD. Rapamycin which can maintain the contractile phenotype of VSMCs may be useful to prevent the formation and progression of TAD.

There are several limitations in the present study. Firstly, the sample capacity in this study is relatively small, leading to the unpredictable confounding factors that might cause unexpected biases. For the following studies, the sample size should be enlarged to exclude those biases. Secondly, the present findings based on *in vitro* studies cannot determine whether PC-1 downregulation is the causative factor or the consequence of TAD. Then, *In vivo* studies were not performed for the lack of specific promoter to construct the animal models of TAD whose contractile and synthetic VSMCs were specifically marked. We have been constructing this animal model based on the Cre-LoxP system which might be favorable for the further studies. Finally, PC-1 is also involved in the hemodynamic regulation of vascular endothelial cells (Besschetnova et al., 2010; Cordova-Casanova et al., 2018), which may also contribute to TAD while was not explored in the present study. To fully understand the molecular mechanisms underlyting TAD pathogenesis, additional functional experiments and the specific animal models are necessary.

## Conclusion

Our study has revealed the decreased expression of PC-1 in TAD. Through activation of mTOR/S6K/S6 signaling pathway, PC-1 downregulation induces aortic VSMCs phenotypic alteration from contractile to synthetic type and promotes ECM dysfunction. The downregulation of PC-1 might be a potential mechanism for the development and progression of TAD.

## Data Availability Statement

The raw data supporting the conclusions of this article will be made available by the authors, without undue reservation.

## Ethics Statement

The studies involving human participants were reviewed and approved by Ethical Committee of Shanghai Chest Hospital. The patients/participants provided their written informed consent to participate in this study.

## Author Contributions

JZ and L-xW designed this study and reviewed the manuscript. FL and WZ performed all the experiments. Y-bH performed the statistical analysis and wrote this article. W-rM and JX collected the normal and TAD aortic samples. All authors contributed to the article and approved the submitted version.

## Conflict of Interest

The authors declare that the research was conducted in the absence of any commercial or financial relationships that could be construed as a potential conflict of interest.
