# Supplementary figures and images for: Polycystin-1 Downregulation Induced Vascular Smooth Muscle Cells Phenotypic Alteration and Extracellular Matrix Remodeling in Thoracic Aortic Dissection
Source: Front Physiol. 2020 Sep 24;11:548055. doi: 10.3389/fphys.2020.548055 (PMC7541897; doi:10.3389/fphys.2020.548055)

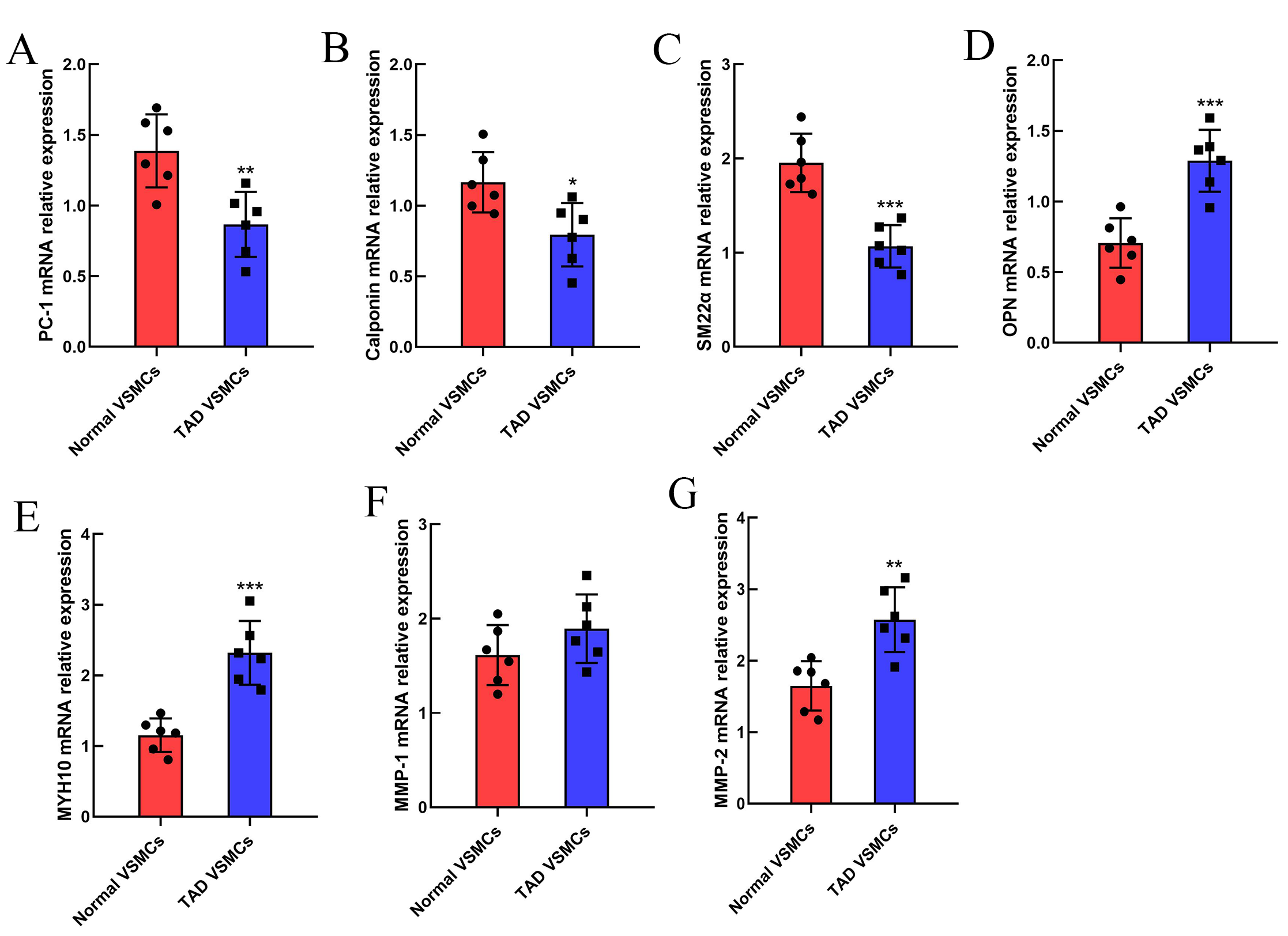

Supplement: Supplementary file 1 [file Image_1.tif]

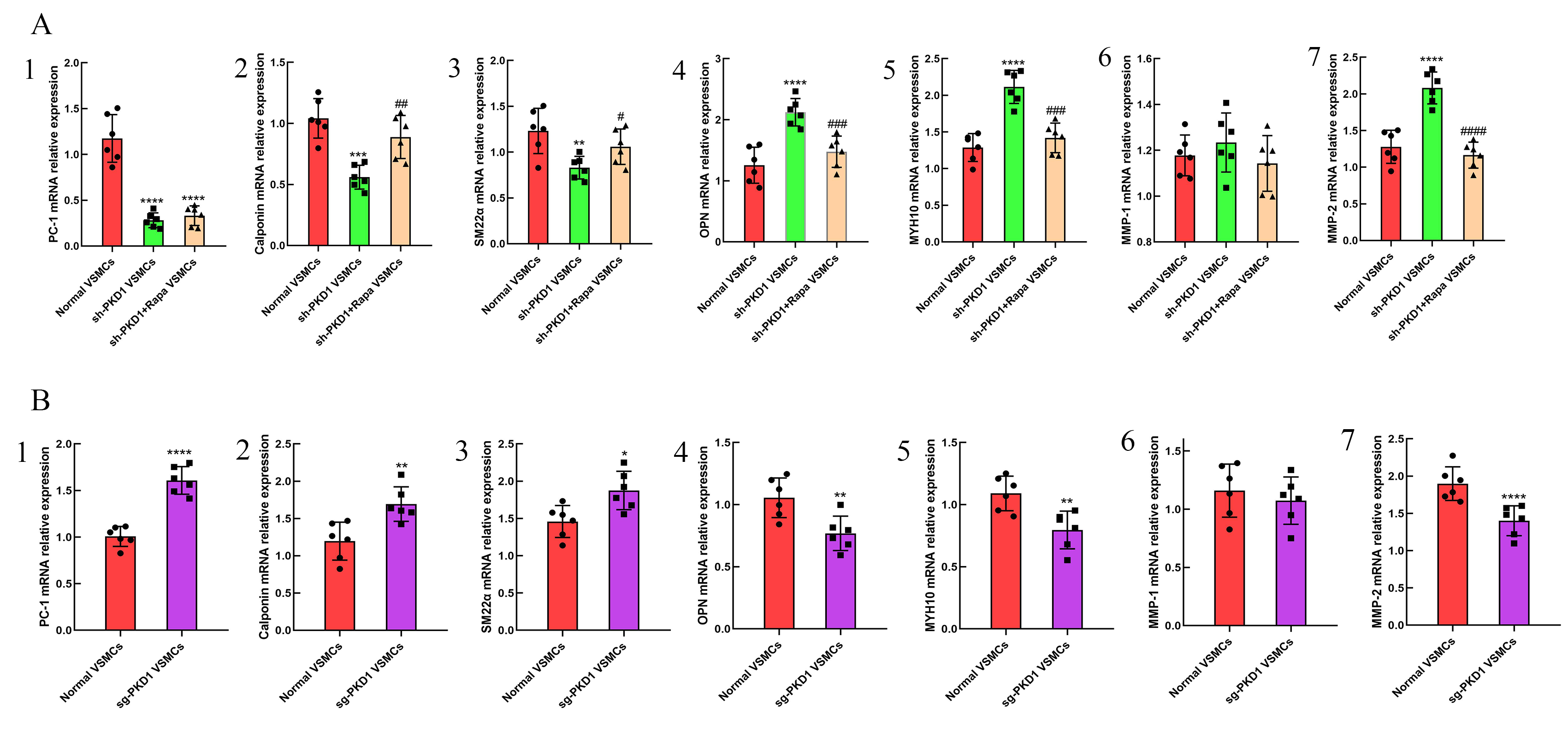

Supplement: Supplementary file 2 [file Image_2.tif]

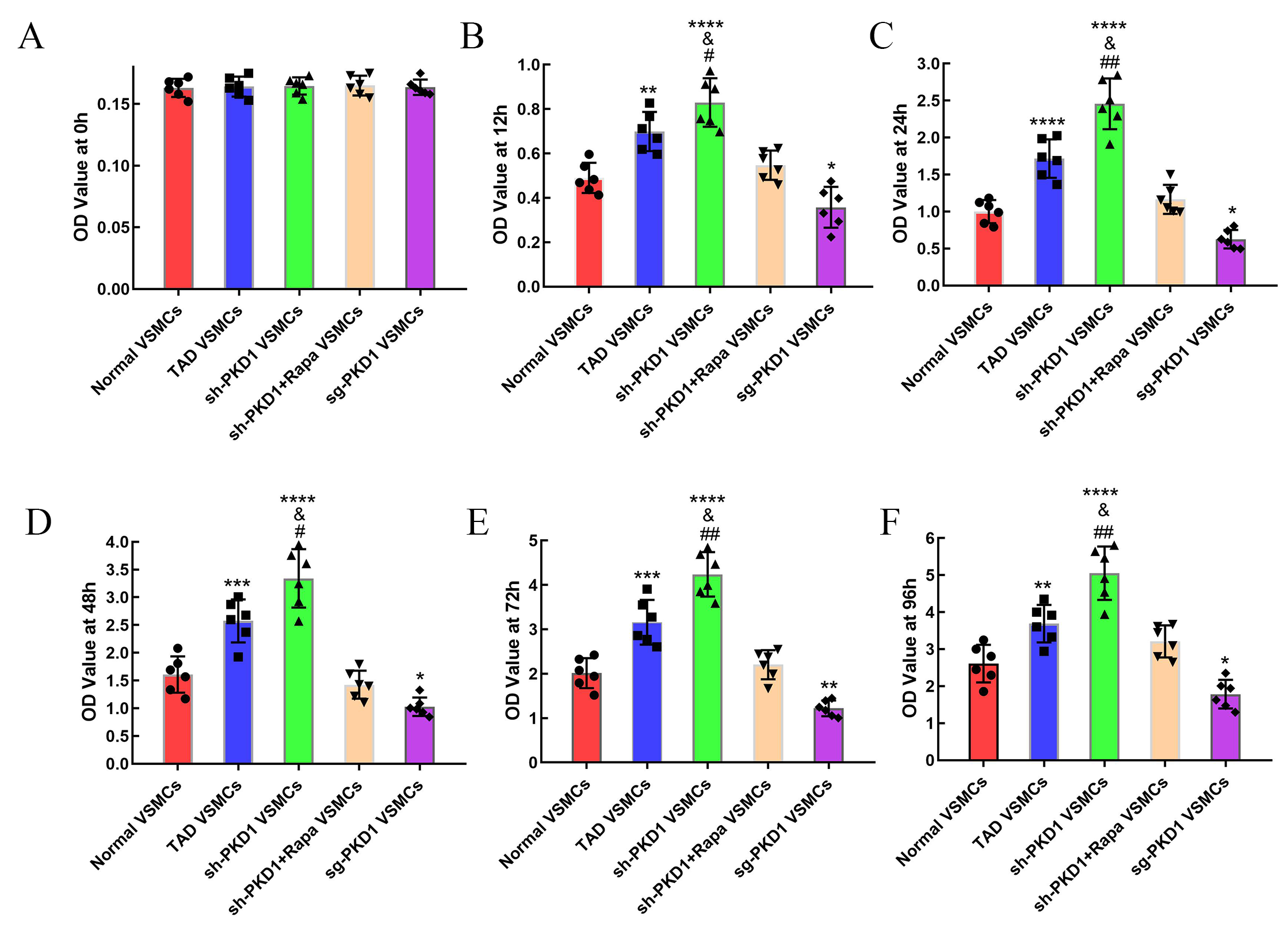

Supplement: Supplementary file 3 [file Image_3.tif]
